# Structural and functional analysis of cellular networks with *CellNetAnalyzer*

**DOI:** 10.1186/1752-0509-1-2

**Published:** 2007-01-08

**Authors:** Steffen Klamt, Julio Saez-Rodriguez, Ernst D Gilles

**Affiliations:** 1 Max Planck Institute for Dynamics of Complex Technical Systems, Sandtorstrasse 1, 39106 Magdeburg, Germany

## Abstract

**Background:**

Mathematical modelling of cellular networks is an integral part of Systems Biology and requires appropriate software tools. An important class of methods in Systems Biology deals with structural or topological (parameter-free) analysis of cellular networks. So far, software tools providing such methods for both mass-flow (metabolic) as well as signal-flow (signalling and regulatory) networks are lacking.

**Results:**

Herein we introduce *CellNetAnalyzer*, a toolbox for MATLAB facilitating, in an interactive and visual manner, a comprehensive structural analysis of metabolic, signalling and regulatory networks. The particular strengths of *CellNetAnalyzer *are methods for functional network analysis, i.e. for characterising functional states, for detecting functional dependencies, for identifying intervention strategies, or for giving qualitative predictions on the effects of perturbations. *CellNetAnalyzer *extends its predecessor *FluxAnalyzer *(originally developed for metabolic network and pathway analysis) by a new modelling framework for examining signal-flow networks. Two of the novel methods implemented in *CellNetAnalyzer *are discussed in more detail regarding algorithmic issues and applications: the computation and analysis (i) of shortest positive and shortest negative paths and circuits in interaction graphs and (ii) of minimal intervention sets in logical networks.

**Conclusion:**

*CellNetAnalyzer *provides a single suite to perform structural and qualitative analysis of both mass-flow- and signal-flow-based cellular networks in a user-friendly environment. It provides a large toolbox with various, partially unique, functions and algorithms for functional network analysis.*CellNetAnalyzer *is freely available for academic use.

## Background

Systems biology aims at a holistic analysis of biological networks. Mathematical modelling plays a pivotal role for this integrative approach. The arguably most common formalism for cellular networks is kinetic modelling, which has been successfully applied to the study of single pathways and networks of moderate size (e.g. [1,2]). However, building dynamic models with high predictive power requires an amount of reliable quantitative data which is often not available in large-scale networks with hundreds of players and interactions. Structural or qualitative (parameter-free) models relying solely on the often well-known network structure provide an alternative approach still capable to gain useful insights in the functioning of these networks [3-6].

*CellNetAnalyzer *(CNA) is a graphical user interface for MATLAB providing a comprehensive toolbox for structural and functional analysis of different types of cellular networks. CNA extends its predecessor *FluxAnalyzer*, originally developed for metabolic network analysis [7], by new methods for signalling and regulatory networks, i.e. for networks where signal flows are dominating (in contrast to mass flows in metabolic networks). Herein, we will give a general overview on CNA with focus on the new functionalities.

## Implementation

**Figure 1:**
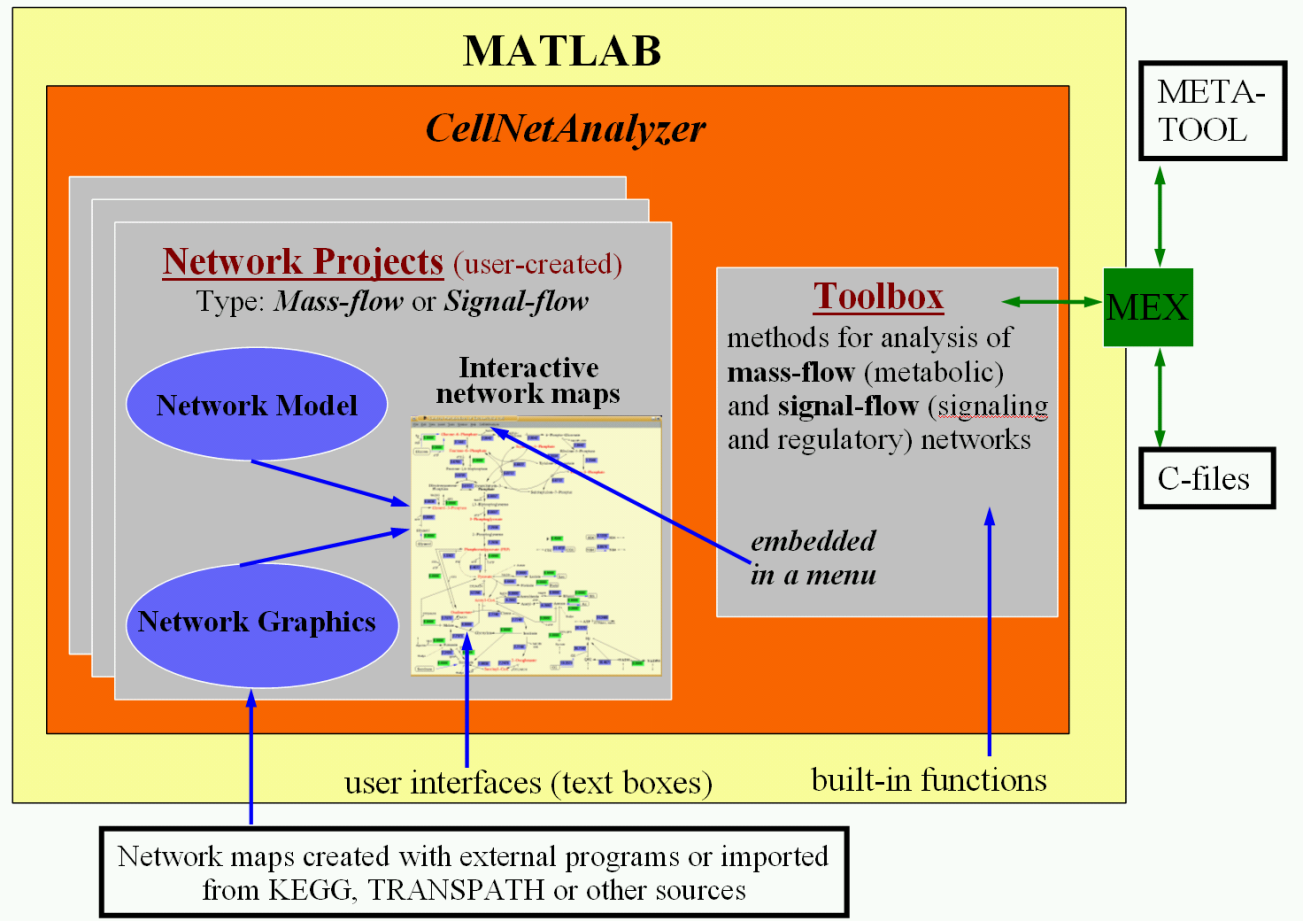
**General set-up of *CellNetAnalyzer***. For explanations see main text.

The general set-up of CNA is shown in Figure 1. CNA is a toolbox for MATLAB ^® ^(Mathworks Inc.), a widely-used software for numerical computations and complex visualisations of numerical data. CNA has been programmed with the MATLAB language enabling to use built-in functions of MATLAB, in particular those for matrix operations. MATLAB also allows to call external C programs via the so-called MEX interface. CNA makes use of this interface for some computationally intensive calculations (see below).

As CNA runs in the MATLAB environment and because MATLAB is available for many operating systems, CNA itself is platform-independent. Upon starting CNA in MATLAB's command window, CNA runs virtually autonomously as a graphical user interface.

### Network projects

**Figure 2:**
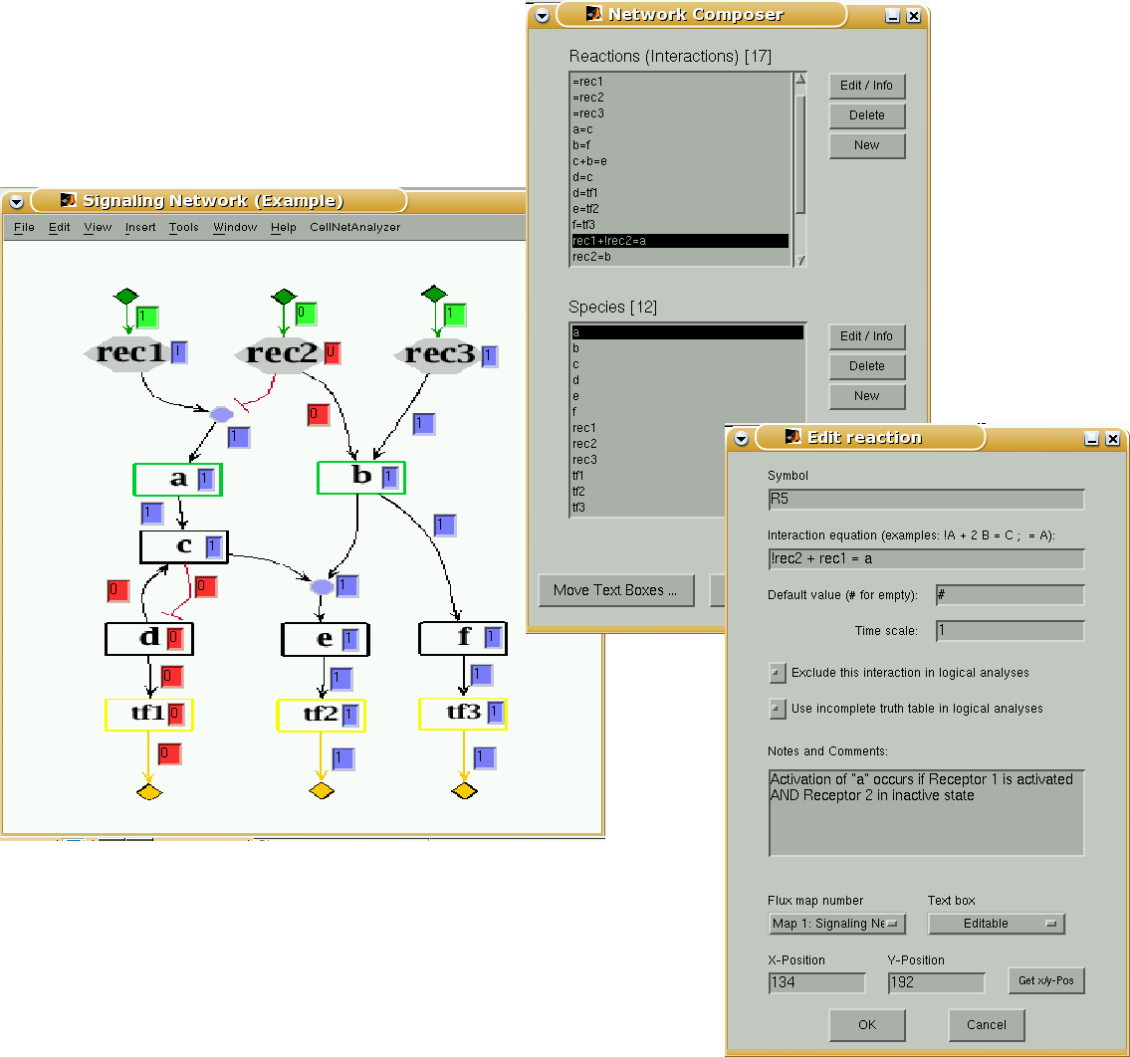
**Example of an interactive network map of a simple signalling network**. (Map and model were created with ProMoT [11] and exported to *CellNetAnalyzer*.) Note that the red edges represent inhibition (NOT operation) and blue circles indicate logical ANDs (compare also with Table 1). In the presented scenario, receptors *rec1 *and *rec3 *are activated by external signals, whereas receptor *rec2 *is not. The text boxes along the (hyper)arcs display signal flows (green boxes: fixed signal prior computation; blue boxes: signal flow activating a species (signal flow is one); red boxes: no activating signal is flowing along this connection). The text boxes attached to the species represent the states of the nodes in the resulting logical steady state. Network composer and the mask for editing reactions/interactions are also shown.

As a fundamental step, CNA facilitates the construction of *network projects *which can represent either a *mass-flow *(stoichiometric, metabolic) or a *signal-flow *(signal transduction/regulatory) network. For both types of networks, the abstract network model can be set-up and edited via a *Network Composer *and input masks (see Figure 2) or, alternatively, via ASCII (text) files. The network description comprises always the declaration of *species *and *reactions *and their respective attributes. The same nomenclature is used for both kinds of networks, but with a different meaning. In mass-flow networks (MFNs), reactions correspond to the stoichiometric conversions. Therefore, the reaction equation

A + B → C     (1)

is interpreted as usual in MFNs, namely that the two reactants A and B are converted into C. A and B are consumed in this process representing the key characteristic of mass flows. MFNs are stored by the stoichiometric matrix and other variables such as the capacity and reversibility constraints of the reactions [5].

In contrast, a reaction (or *interaction *in this context) in signal-flow networks (SFNs) expresses how the "product" (end node) of the reaction can be *activated *by the "reactants" (start nodes). Accordingly, equation (1) means in SFNs, that C becomes activated if A AND B are active, i.e. "+" is interpreted as a logical AND. As a key characteristic of signal flows, the start nodes A and B on the left-hand side of the reaction equation are not necessarily consumed in this process. For example, eq. (1) could express that C becomes activated (e.g. phosphorylated at two phosphorylation sites) if the two kinases A and B are in an active state. Accordingly, in CNA, an SFN is a logical (Boolean) network, where each reaction represents a Boolean AND clause expressing a condition under which the end node of this reaction becomes activated. If several reactions point at one species *S*, then they are OR'ed together giving eventually rise to a Boolean function determining the activation state of *S*. Accordingly, in the example network shown in Figure 2 and Table 1, node *c *becomes active if *a *OR *d *are active. In addition to AND and OR, representing arbitrary Boolean functions requires a NOT operator (indicated by "!"). Furthermore, multi-valued logic is possible in CNA. Therefore, the equation

3 A + !B → 2 C     (2)

means that "C reaches level 2 if A is at level 3 AND B is inactive (level 0)". Using the formalism described above, CNA represents the logic of SFNs as *logical interaction hypergraphs *(strongly related with the sum-of-product or disjunctive normal form (DNF) representation of Boolean functions) which can be conveniently stored in two matrices, each having as rows the species and as columns the interactions [8]: an *interaction matrix *captures the logical coefficients (similar as the stoichiometric matrix the stoichiometric coefficients) and a *NOT-matrix *stores where a NOT operation occurs.

*Interaction graphs *(signed directed graphs where each arc connects one start with one end node indicating a causal positive or negative dependency) can also be encoded in this formalism: graphs are treated as special cases without AND connections. In these cases, the interaction matrix coincides with the incidence matrix of this graph.

Specifically for SFNs, CNA also supports a *time-scale *attribute as well as *incomplete truth tables *for handling interactions with uncertain logical concatenation [8].

**Figure 3:**
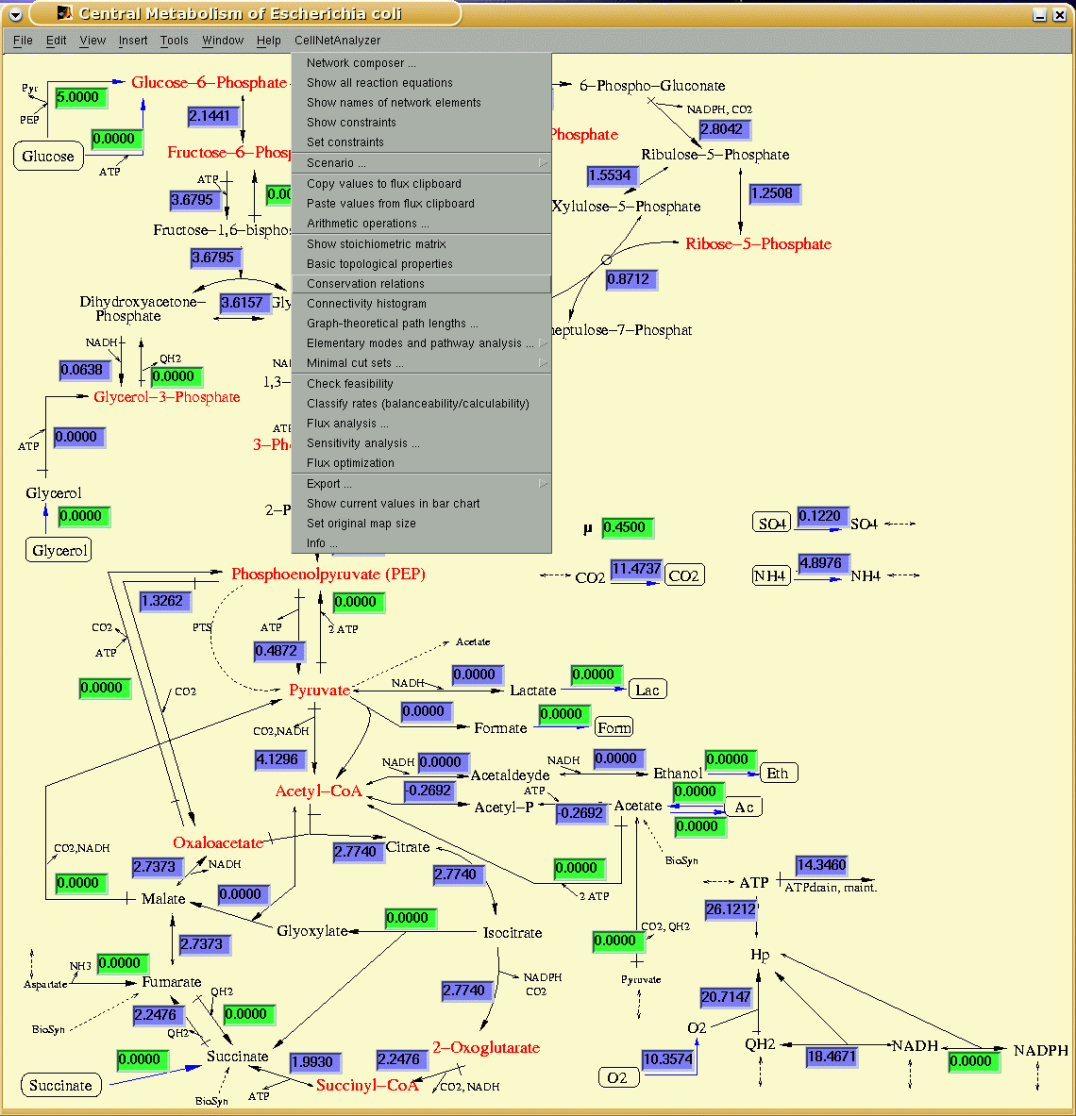
**Example of an interactive network map of a mass-flow (metabolic) network in *CellNetAnalyzer***. The network shows the central metabolism of *E. coli *studied in [9]. The text boxes display a computed steady-state flux distribution (the numbers represent reaction rates). Green boxes indicate rates fixed prior computation and blue boxes show calculated rates. The pull-down menu of *CellNetAnalyzer *(for mass-flow networks) is also shown.

**Figure 4:**
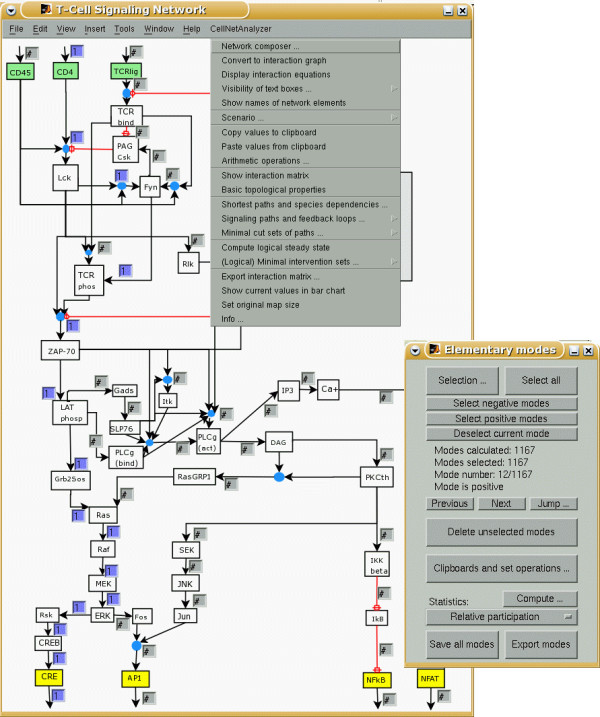
**Example of an interactive network map in *CellNetAnalyzer*, here from a signal-flow network related to signalling pathways in T-cells**. The blue text boxes display a signalling path from the receptor CD4 to the transcription factor CRE. The menu of *CellNetAnalyzer *(here for signal-flow networks) and the control panel for elementary modes/signalling paths are also shown.

In principle, network analysis with CNA can be done without any graphical representation of the network. However, for visualisation purposes, each network project can be assigned one or several network maps (Figures 1, 2, 3, 4). Network maps must be provided by the user, either created with the help of external drawing programs (e.g. CorelDraw) or by using maps available at databases such as KEGG or TRANSPATH. Thus, network maps in CNA are static but offer a high flexibility regarding their design. The link between network maps and network model is realised in CNA by text boxes, which are small user interfaces, each associated to one network element. They can be positioned via drag-and-drop at a proper place on the maps and enable input and output of context dependent data. In CNA, a network map with text boxes is called *interactive network map*. Three examples are shown in Figures 2, 3, 4: Figure 2 displays a simple hypothetical SFN whose reactions are given in Table 1, Figure 3 shows a MFN project for the central metabolism of *E. coli *(studied in [9]), and Figure 4 displays a SFN model of signalling pathways involved in T-cell activation (a larger version of this model is studied in [10]). Notice the different visual representation of these three networks: CNA does not impose any restrictions on the design of these maps what is possible because the maps can be created with arbitrary (external) drawing tools.

An elegant solution for setting up logical network models is provided by a new feature of the modelling tool ProMoT: the model is created in a visual environment, and both the map and the underlying network can be exported to CNA and other formats [11]. ProMoT is a stand-alone, object-oriented tool to set up modularly and hierarchically structured models of technical and biological systems in a visual manner by a simple drag-and-drop procedure [12]. A new library containing logical elements (compounds, NOTs, ANDs, etc.) has been developed which allows to set up models according to the sum-of-product formalism as used in CNA. Properties such as the initial value and time scale can be added via a text menu. Once finished, the models can be exported: one obtains a map (as an image file) and CNA text files defining the mathematical model. Importantly, the corresponding positions of the textboxes required for the interactive network maps in CNA are also included. For more details see [11] and ProMoT's web-site [13]. As an example, the network project shown in Figure 2 has been produced with the new features of ProMoT.

## Results and Discussion

CNA provides a powerful battery of methods for *functional network analysis*, i.e. for characterising functional states, for detecting functional dependencies, for identifying intervention strategies, or for giving qualitative predictions on the effects of perturbations. A typical scenario is to check "whether and how a certain metabolite (transcription factor) can be synthesised (activated) in a metabolic (signalling) network under a certain knock-out condition with a given set of external resources (input stimuli)". The user may start computations from a pull-down menu whose content depends on the type of the network project (mass-flow or signal-flow; see Figures 3 and 4). All functions are described in detail in the CNA user's manual, here we shall only give an overview and emphasise novel routines, in particular for signal-flow networks.

### Metabolic networks

Regarding mass-flow networks, the majority of methods implemented in CNA belong to the constraint-based approach frequently used for metabolic network analysis [4,5]. Additionally, some methods for graph-theoretical analysis are provided. The main features are:

• general topological properties: (dead ends, blocked reactions, parallel reactions, enzyme subsets, etc)

• (elementary) conservation relations

• graph-theoretical features: shortest path lengths, connectivity analysis, network diameter etc.

• metabolic flux analysis: computing steady-state flux distributions from a set of given reaction rates (see example in Figure 3); handling redundant systems including gross error detection; feasibility check of flux scenarios

• flux balance analysis: find optimal flux distributions for arbitrary linear objective functions

• metabolic pathway analysis with elementary modes

• minimal cut set analysis: intervention strategies for repressing a certain functionality in the network

Most of these functions were already part of *FluxAnalyzer *[7]. The tools provided for a comprehensive analysis of elementary modes (EMs) and minimal cut sets (MCSs) are a particular strength of CNA and have been revised and algorithmically improved. EMs represent the minimal functional units (pathways) of a metabolic network [14], whereas minimal cut sets (MCSs) can be seen as minimal failure modes [15,16]. EMs and MCS are actually dual descriptions of a network's functionality [16], each providing different applications. In particular EM analysis has become a standard tool in metabolic network analysis [14,5]. However, the inherent combinatorial complexity makes the calculation of EMs and MCSs in large networks a computationally hard task. CNA offers state-of-the-art algorithms and uses the MEX interface of MATLAB to call (faster) external C-files [17] (see Figure 1). In particular, CNA provides an interface to Metatool [18] enabling to compute EMs on the fly with the probably fastest algorithm currently available. The computation of MCSs has been revised; it relies now on the *Berge *algorithm known from the theory of minimal hitting sets [19,16] outperforming the original algorithm introduced in [15] by about two orders of magnitudes.

Apart from displaying EMs and MCSs directly in the interactive maps, CNA facilitates a detailed statistical assessment of large sets of MCSs and EMs. An important feature is the opportunity to select subsets of EMs or MCSs by specifying a set of criteria (e.g. "select all EMs involving reaction R1 but not R2"). Then, statistical properties can be calculated for the current selection and compared with other selections, useful e.g. to assess the importance of a reaction under different growth conditions. Such calculations include (relative) reaction participation, structural couplings, or optimal product yields.

### Signalling and regulatory networks

CNA provides new algorithms designed for a functional analysis of signal-flow networks (most of the implemented methods were detailed in [8]). Basically, each function operates either directly on the logical network model of the SFN or on the underlying interaction graph. The latter can be derived automatically from the logical hypergraph representation by splitting all the AND connections. For example, the reaction in eq. (2) would be decomposed into one positive (A→C) and one negative arc (B→C).

#### Interaction graphs

The main features of CNA for studying interaction graphs comprises the computation and analysis of:

• general graph-theoretical network properties

• signalling paths and feedback loops (circuits)

• distance matrices capturing the lengths of the shortest negative/shortest positive path between all ordered pairs of species

• the *dependency matrix*

General graph-theoretical properties that can be computed include the number of components, the network diameter and others. A more sophisticated feature is the computation of signalling paths and of the network's feedback loops (circuits). For example, one can compute all (directed) signalling paths connecting a species *i *with a species *k *each representing a path along which *i *can influence *k*. Feedback loops represent sub-networks along which a species *k *can influence itself (without visiting a node twice except *k*). They govern network dynamics and stability and are the driving force of fundamental physiological process such as differentiation, oscillations or homeostasis [20,21]. A sign indicating a positive (even number of involved negative arcs) or negative (odd number) overall influence is assigned to each path and circuit.

For computing paths and circuits, CNA utilises the same algorithmic approach as for EMs in metabolic networks [8]. The only difference is that each path and circuit gets an overall sign. As for EMs in MFNs, paths and circuits can be displayed in the maps and statistically assessed (see Figure 4). Furthermore, again in close analogy to metabolic networks, MCSs interrupting e.g. a given set of feedback loops or/and paths can be computed. This feature can also be used to decompose a network into monotone dynamical sub-systems in the sense as discussed in [22].

**Figure 5:**
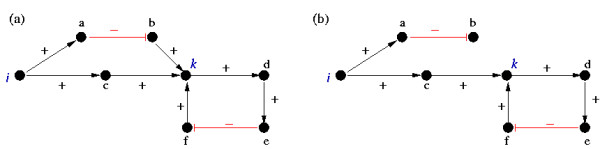
**Two examples of simple signed directed graphs (interaction graphs)**. Graph (b) is the same as graph (a) except that the negative arc from *b *to *k *has been removed.

In very large networks, a full enumeration of paths and circuits becomes infeasible since the number of these objects depends exponentially on the network size. Instead of computing *all *paths and circuits, one may then focus on the *shortest *paths and *shortest *circuits, whose computation is much less expensive but still provides important functional insights. Shortest paths algorithms are well-known from graph theory (e.g. Dijkstra algorithm [23]), however, interaction graphs are signed directed graphs and a special procedure is required for computing the length of the *shortest positive *and of the *shortest negative *path from a node *i *to a node *k*, denoted with P_ik _and N_ik_, respectively. Here we also allow *i *= *k*, i.e. P_kk _and N_kk _represent the length of the shortest positive/negative circuit involving *k*. Note that P_ik _and N_ik _are usually different (sometimes only one of both is finite) and the minimum of both gives the (unsigned) shortest path length. For example, in Figure 5(a), we have P_ik _= 2, N_ik _= 3, N_kk _= 4 and P_kk _= ∞ (no positive circuit running over *k *exists). In Figure 5(b), one arc has been removed implying N_ik _= ∞ (a negative path from *i *to *k *does no longer exist, i.e. the length is infinite).

The importance of shortest positive/negative paths and circuits has been emphasised in [8], however an algorithmic scheme for computing them has neither been given in this reference nor could the authors find one in the literature. The algorithm implemented in CNA extends the Dijkstra algorithm and determines simultaneously P_ik _and N_ik _for all ordered pairs of nodes (*i,k*). Again, *i *= *k *is also considered, i.e. shortest positive/negative feedback circuits are computed concurrently. In the *j*-th iteration, all the P_ik _and N_ik _of length *j *are identified using the shortest paths identified in iteration *j*-1. For example, in Figure 5(a), in the 2^nd ^iteration, a path length of 2 would be found for N_ib_, P_ik_, N_ak_, P_bd_, P_cd_, P_ke_, N_df_, N_ek_, P_fd_. Hereby it is important to keep track of the predecessor node *p *of *k *through which the shortest path (separately for positive and negative path) from *i *is running before reaching *k*. For example, in Figure 5(a), we would store node *c *for P_ik_, node *b *for N_ik_, node *a *for N_ib _and so on. This information can be used to reconstruct, at the end, the determined shortest path from *i *to *k *(by travelling back from *k *to *i *along the predecessor nodes). Moreover, as a key feature of our algorithm, this information is required to check for each shortest path candidate (again, by travelling back along the predecessor nodes) that no circuit is contained. In usual shortest path algorithms, this can not happen but due to the parallel determination of P_ik _and N_ik _it may here. For example, in Figure 5(b), a positive path from *i *to *k *exists (P_ik_= 2) and *k *is a node in a negative circuit. The algorithm finds first the positive path from *i *to *k*. Then it will further look for a negative path and while running through the negative circuit, it would find, in total, a negative *walk *from *i *to *k *which is, however, not a path in the graph-theoretical sense (*k *is visited twice) and has therefore to be discarded.

**Figure 6:**
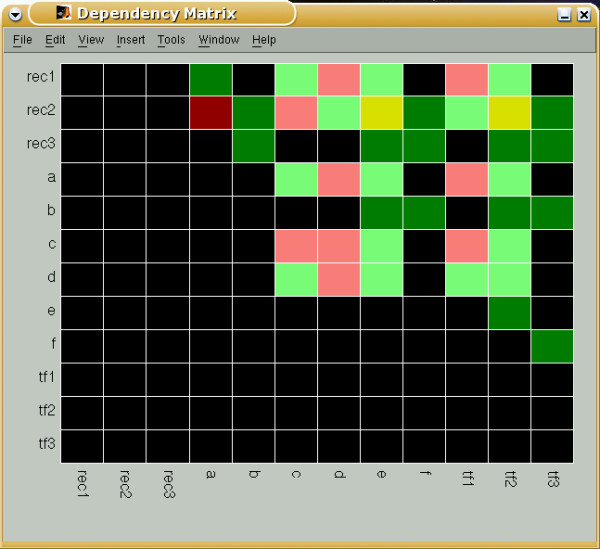
**Dependency matrix of the network in Figure 2 as displayed by *CellNetAnalyzer***. The color of a matrix element M_xy _has the following meaning (see [8] and text): (i) dark green: x is a strong (total) activator of y; (ii) light green: x is a weak (non-total) activator of y; (iii) dark red: x is a strong (total) inhibitor of y; (iv) light red: x is a weak (non-total) inhibitor of y; (v) yellow: x is an ambivalent factor for y; (vi) black: x does not influence y.

With the algorithm described above, all the shortest positive/negative paths and circuits can be computed efficiently in large-scale networks (within seconds). They not only provide distance measures, but also show (i) whether *any *positive or/and negative path between a pair of nodes or (ii) whether any positive/negative circuit involving a certain species exists at all. For example, in Figure 2, there is a positive path from *rec3 *to *tf2 *and *tf3 *and no negative path to any of the sink nodes ("transcription factors") *tf1, tf2, tf3*. From *rec2 *all sink nodes can be reached via a positive path, and *tf2 *also along a negative one. Species *c *and *d *are the only ones involved in a (negative) feedback loop. Such relationships, classified for all pairs of nodes, are represented by CNA in the so-called *dependency matrix *[8] which reveals network-wide interdependencies (Figure 6 shows the dependency matrix displayed by CNA for the network in Figure 2). Each row of the dependency matrix characterises how the corresponding species influences the other nodes, whereas each column indicates how the corresponding species *is *influenced by the others. Intuitively, *i *is called an activator of *k *if P_ik_<∞ and N_ik _= ∞ (there are only positive paths from *i *to *k*) and marked as an inhibitor of *k *if P_ik _= ∞ and N_ik_<∞ (all paths from *i *to *k *are negative). More precisely, the dependency matrix as introduced in [8] characterises for each ordered pair of nodes (*i*,*k*) the effect of *i *on *k *by one of the 6 following cases (D_ik _denotes the distance between *i *and *k*, i.e. the minimum of P_ik _and N_ik_):

• *i *has *no effect *on *k *if P_ik_= N_ik _= ∞, i.e. D_ik _= ∞ and there is no path from *i *to *k *(example: *rec3 *has no effect on *tf1 *in Figure 2)

• *i *is a *strong *(or *total*) *activator *of *k *if P_ik_<∞ and N_ik _= ∞ and there is no node *z *such that D_iz_<∞ and D_zk_<∞ and N_zz_<∞ (example: *rec3 *is a strong activator of *tf2 *and *tf3*)

• *i *is a *weak (or non-total) activator *of *k *if P_ik_<∞ and N_ik _= ∞ and there is a node *z *such that D_iz_<∞ and D_zk_<∞ and N_zz_<∞ (example: *rec2 *is a weak activator of *tf1*)

• *i *is a *strong (or total) inhibitor *of *k *if P_ik _= ∞ and N_ik_<∞ and there is no node *z *such that D_iz_<∞ and D_zk_<∞ and N_zz_<∞ (example: *rec2 *is a strong inhibitor for *a*)

• *i *is a *weak (or non-total) inhibitor *of *k *if P_ik _= ∞ and N_ik_<∞ and there is a node *z *such that D_iz_<∞ and D_zk_<∞ and N_zz_<∞ (example: *rec1 *is a weak inhibitor of *tf1*)

• *i *is an *ambivalent factor *for *k *if P_ik_<∞ and N_ik_<∞ (example: *rec2 *is an ambivalent factor for *tf2*)

The global dependencies collected in the dependency matrix facilitate valuable qualitative predictions about the effects of perturbation or knock-out experiments [8]. For example, the effect of strong activators and strong inhibitors is strictly monotone. Thus, starting from a resting state, increasing the level of (active) *rec3 *should lead to an increase in the activation level of *tf2 *and *tf3 *since *rec3 *is a strong activator of *tf2 *and *tf3*. In contrast, it should have no effect on *tf1 *since there is no connection from *rec3 *to *tf1*. Predictions on the effects of *weak *activators and *weak *inhibitors are more limited due to the involvement of negative feedback loops, however, we can at least predict that there exists a time window (of unknown length) where the affected species can only increase (weak activators) or decrease (weak inhibitors) after a (positive) perturbation. Only for ambivalent factors nothing at all can be said regarding perturbation effects since then positive and negative influence paths are competing and the overall effect will depend on kinetic parameters and concentrations.

Note that this type of analysis is related to the theory on monotone systems and the notion of consistent graphs [24,22].

In general, when computing (all or shortest) paths and feedback loops, CNA allows the user to exclude nodes or edges for testing knock-outs effects.

#### Logical networks

Boolean or logical networks have been extensively used for modelling small or medium-scale (gene) regulatory networks, typically characterised by having few (or no) inputs but many feedback circuits [20,25-28]. Main aspects that have been studied focus on the discrete dynamics of the system including its attractors, global stability, and the potential transition paths. GINsim, a recently developed software tool, supports this type of analyses [28]. Although the functions provided by CNA for logical network analysis are in principle applicable to gene regulatory networks, they are especially useful in networks which are structured in input, intermediate, and output layer as typical in signal transduction networks [8]. Logical analysis in CNA aims to a characterisation of the input/output behaviour of the system and to search for interventions that can change the natural behaviour into a desired one:

#### (1) Logical steady states

CNA computes the logical steady state that follows from a user-defined scenario (consisting of a set of input stimuli, e.g. receptor X is activated and receptor Y not). This functionality enables to study how signals are propagated through the network and how a network responses to certain stimuli [8]. Again, the user may fix some signal flows or states (off/on) mimicking deactivation (e.g. by inhibitors or knock-outs) or permanent activation, respectively. In Figure 2, such a scenario is displayed: receptors *rec1 *and *rec3 *were considered to be activated and *rec2 *not. The logical steady state was then computed showing the response of the network elements to this activation pattern.

Note that, sometimes, the logical steady state resulting from a given input pattern may be not unique for all nodes or a logical steady state does even not exist [8]. In such a case, CNA will indicate for which compounds a unique logical steady state can not be determined.

#### (2) Minimal intervention sets

CNA provides a complex routine for computing (logical) minimal intervention sets (MISs; [8]). Similar to MCSs in metabolic networks, a MIS is a minimal set of interventions by which a user-defined intervention goal (e.g. the permanent activation/deactivation of certain compounds) will be satisfied. The user may define (i) an intervention goal by setting desired on/off values for the respective states and signal flows and (ii) a scenario (e.g. a pattern of inputs) to which the network is exposed. CNA searches then for combinations of interventions so that the resulting logical steady state will satisfy the intervention goal. In contrast to minimal *cut *sets, an intervention may represent not only the permanent deactivation (knock-out) but also a permanent activation (e.g. knock-in mutation) of a compound.

To illustrate the concept of MISs consider Figure 2: assume we want to have transcription factors *tf1 *inactivated and *tf2 *activated (and don't care about *tf3*). In total, there are 21 MISs. One example is {*rec1 *= 1, *rec2 *= 0, *rec3 *= 1} which indicates (the only) set of input stimuli that would satisfy the intervention goal. Another one is {*a *= 1, *rec2 *= 1}, where *a *and *rec2 *are permanently hold in an active state (e.g. by knock-in mutation). Here, the inputs at *rec1 *and *rec3 *are irrelevant for achieving the intervention goal.

For computing MISs, CNA uses an almost brute-force approach, since it checks systematically all *minimal *combinations of interventions (first of size 1, then size 2 and so on) whether they lead to a fulfilment of the intervention goal when the network reaches the logical steady state. However, some important heuristics are exploited. For example, only those species are subject to interventions that have an influence on compounds being part of the intervention goal (can be checked quickly via the dependency matrix). As one is typically interested in the (small) MISs with only few interventions and because the computation of MISs with higher cardinality is the most time-consuming part, CNA allows to set a maximum cardinality.

MISs can be displayed in the maps and assessed statistically. As outlined in [8] and demonstrated in [10], MISs provide a powerful tool for analysing signalling networks. Some applications are:

(i) searching for intervention strategies for repressing/provoking certain behaviours.

(ii) identifying fragile points in the network and estimating the importance of network elements for different functions (example: activated *b *is mandatory for getting *tf2 *and *tf3 *activated in the network in Figure 2)

(iii) identifying failure modes which might cause an observed abnormal (pathological) behaviour of the network (example (Figure 2): if *tf1 *is on and *tf2 *off in experiments under all possible combinations of input stimuli then a failure (e.g. caused by a mutation) in node *c *is likely since {*c *= 0} is a MISs for the intervention goal *tf1 *= on and *tf2 *= off)

(iv) searching for candidates of missing links in the network by which experimental data not consistent with the current network model could be explained (for examples see [10])

### Additional features

A number of features, most of them available for both types of network projects, make work with CNA easier. *Scenarios*, e.g. representing flux distributions or a set of logical states and signal flows, can be saved and then later reloaded. A clipboard enables to store the currently displayed scenario temporarily in memory; it can be pasted back afterwards or compared with other scenarios. The size, context-dependent colours and visibility of the text boxes (see Figures 2, 3, 4) are definable by the user. Mass-flow networks may be exported in ASCII (plain text) format (stoichiometric matrix, names of species and reactions) as well as imported and exported in SBML format. In signal-flow networks, the interaction/incidence matrix and names of species and reactions can be exported in ASCII format. Note that exchange of logical models is not supported (yet) by SBML.

### Integrating signal-flow and mass-flow networks

An important issue towards a holistic analysis of cellular networks is network integration, i.e. to facilitate the analysis of networks with mass *and *signal flows in one coherent topological model. This would enable to relate events in the metabolism with events in signaling and regulatory networks. The key question is how to design conceptually the interface for connecting signal flows with mass flows and vice versa. Some approaches have been proposed in the literature. Covert et al. [28] connected a regulatory network, controlling the activity of some metabolic pathways, with a metabolic network model. The regulatory network, hierarchically on top of the metabolic model, was represented as a Boolean network whose inputs are the external conditions (e.g. substrate and oxygen availability) and whose outputs are the reactions to be switched on or off in the metabolic (stoichiometric) network model. This type of modelling is possible in CNA and requires only one intermediate step: the regulatory (as SFN) and the metabolic network (as MFN) are represented in two separate models. The inputs of the regulatory network are the environmental conditions and the outputs of the regulatory network are the states (on/off) of the reactions in the metabolic network. Defining a given set of stimuli in the regulatory model will result in a corresponding logical steady state. Then, one exports the reactions with state 0 (with "Save scenario"). By using the same reaction identifiers in the metabolic model, this scenario can be loaded showing all the reactions in the metabolic network which have been switched off (all others are potentially available). Then, calculations such as elementary modes or optimal flux distributions can be conducted.

Though this approach is useful for a number of applications, it is unidirectional and is not able to close the loop, i.e. to account for the different kinds of interactions going from the metabolic network back to the regulatory or signalling part.

A quite different approach for connecting mass-flow with signal-flow networks, relying on interaction graphs, has been introduced recently [30]. At least some of the techniques proposed in this work can already be employed with CNA in a straightforward manner by using the functions provided for interaction graphs. Furthermore, in [31] another method was introduced called "network expansion" which relies on a Boolean description of metabolic networks and might have the potential to integrate also signalling networks. Again, this approach is already supported by CNA since network expansion relies on computing logical steady states in Boolean networks [8].

In our opinion, all of the above mentioned approaches enable the analysis of specific features of integrated mass-flow/signal flow networks but seem not yet general enough to consider all of the potential types of interactions that may occur. Accordingly, conceiving a more general conceptual framework for combining signal and mass flows and implementing it in CNA is a major aspect of our future work.

## Conclusion

An increasing number of software tools is available for Systems Biology (see e.g. [32]). Some of them are devoted to topological or qualitative analysis of cellular networks, including Metatool [17] for metabolic and GINsim [28] for gene regulatory networks.*CellNetAnalyzer *is a single suite to perform structural and functional analysis of both mass-flow- and signal-flow-based cellular networks in a user-friendly environment. CNA exploits that stoichiometric networks, (interaction) graphs and logical networks can all be represented internally as hypergraphs, albeit the methods for analysing these networks have then to be chosen according to the type of flows that are carried by the reactions (i.e. by the hyperarcs). CNA offers a comprehensive toolbox with various, partially unique, functionalities and algorithms for analysing both types of networks. CNA (and its predecessor *FluxAnalyzer*) has been downloaded by more than 600 independent researchers world-wide. Recently, the new functions for signal-flow networks have been successfully applied to a large-scale logical model of signalling pathways involved in T-cell activation [10], comprising 94 compounds and 123 interactions. Using the methods implemented in CNA, this model was able to provide deeper insights in the functioning of the signalling network governing T-cell activation and to unravel important and previously unknown aspects of this complicated process.

## Availability and Requirements

*CellNetAnalyzer *requires MATLAB^® ^version 6.1 or higher. For a few calculations, the MATLAB Optimisation toolbox is required.

For academic purposes,*CellNetAnalyzer *including its manual can be obtained for free via the web-site



Commercial licenses are available for non-academic users.

## List of abbreviations used

CNA CellNetAnalyzer

MFNs mass-flow networks

SFNs signal-flow networks

EMs elementary modes

MCSs minimal cut sets

MISs minimal intervention sets

## Authors' contributions

SK developed *CellNetAnalyzer *and conceived and implemented the algorithms. JSR tested and applied *CellNetAnalyzer *and gave proposals for new features. EDG gave suggestions for improvements of CNA. All authors read and approved the final manuscript.

## Figures and Tables

**Table 1:** Logical equations of the simple signal-flow network shown in Figure 2.

==> rec3 ==> rec2 ==> rec1 !rec2 + rec1 ==> a rec2 ==> b rec3 ==> b a ==> c c + b ==> e d ==> c b ==> f !c ==> d f ==> tf3 d ==> tf1 e ==> tf2 tf3 ==> tf2 ==> tf1 ==>

A "+" indicates an AND and a "!" a NOT operator.
